# Acknowledgement to Reviewers of *Brain Science* in 2019

**DOI:** 10.3390/brainsci10010047

**Published:** 2020-01-15

**Authors:** Brain Science Editorial Office

**Affiliations:** MDPI, St. Alban-Anlage 66, 4052 Basel, Switzerland

The editorial team greatly appreciates the reviewers who have dedicated their considerable time and expertise to the journal’s rigorous editorial process over the past 12 months, regardless of whether the papers are finally published or not. In 2019, a total of 391 papers were published in the journal, with a median time to the first decision of 18 days and a median time from submission to publication of 36 days. The editors would like to express their sincere gratitude to the following reviewers for their generous contribution in 2019:
Abbasi Layegh, MahmoodLin, Wei-YongAbiri, RezaLin, Yuan-ChiAbutarboush, RaniaLin, Yuan-PinAccolla, Ettore ALindroth, HeidiAdams, James DavidLindsay, AngusAdamson, David CoryLiou, MichelleAghajani, HalehLisi, LuciaAgrawal, ArpanaLitofsky, Norman ScottAguillon-Hernandez, NadiaLiu, Wei-MinAhlfors, SeppoLizarraga, Karlo J.Ahn, Hee-DonLombardi, AngelaAlanio, AlexandreLopez-Escamez, Jose A.Albayram, OnderLópez-López, DanielAldskogius, HåkanLópez-Martínez, AlbertoAlhes, TimLu, Chia-FengAllen, PhilipLubetzky, Anat V.Altschuler, RichardLuftig, RonaldÁlvarez-Sánchez, NuriaLui, FaustaAl-Zaghal, AbdullahLuis Contreras-Vidal, JoseAmianto, FedericoLukoševičius, SauliusAmodeo, Katherine D.Lundervold, Astri J.An, Seong Soo A.Lutfy, KabirullahAnandhan, AnnaduraiLutski, MiriamAnastasi, AlessandraLynch, GeorginaAnderson, JohnLyukmanova, Ekaterina N.Andica, ChristinaMa, WeiyiAndré, NathalieMäättä, SaraAndreu-Sánchez, CeliaMachingura, TawandaArizpe, Joseph M.Maciukiewicz, MalgorzataArnarsson, ArsaellMaciver, BryceAryani, ArashMackisack, MatthewAscoli Marchetti, AndreaMackowiak, MarzenaAssenza, GiovanniMagne, CyrilleAttaheri, AdamMahajan, AbhimanyuAtwood, Brady K.Maher, PamelaAudiffren, MichelMains, Richard E.Avignone, ElenaMaitas-Guiu, JordiBaburamani, Ana A.Makovski, TalBacchelli, ElenaMakowski, NathanBadadani, MallikarjunMalik, RavinderBailey, Neil W.Malkemper, PascalBajic, Dusica Malva, João O.Baker, Kathryn D.Mandracchia, Jon T.Baker, MichaelMandyam, ChitraBakulski, Kelly M.Mansuri, ShahidBalas, BenjaminMantadakis, ElpisBaldermann, Juan CarlosMaoz, UriBaldominos, AlejandroMarano, MassimoBalgiu, Beatrice AdrianaMarceglia, SaraBanerjee, AnweshaMarkant, JulieBanerjee, KalpitaMarquez, GonzaloBarbosa, Flávia LeãoMartelletti, PaoloBarcelo, FransiscoMårtensson, JohanBarkauskienė, RasaMarti, MatteoBarløse, MadsMartinez, AlejandroBarnett, AlexanderMartínez-Banaclocha, Marcos ArturoBarresi, GiacintoMartínez-Hinarejos, Carlos-D.Barson, JessicaMartini, DougBasak, SujitMartin-Pena, AlfonsoBasar-Eroglu, CananMartins, Andre F.Basavarajappa, Balapal S.Martinuzzi, AndreaBasirat, AnahitaMassaro, SebastianoBath, Kevin GMatheson, BrittanyBat-Pitault, FloraMatynia, AnnaBauer, ClemensMavridis, IoannisBayin, N. SumruMaxwell, Jessie R.Bayless, SarahMayr, WinfriedBazán, EulaliaMcAuley, DevinBeaglehole, BenMcCary, Lindsay M.Beasley, ShannonMccully, Kilmer S.Beaudet, Arthur L.McDonough, Ian M.Bechter, KarlMcKenna, Peter EdwardBedore, LisaMehta, AmbereenBelayat, HossainMeloni, BrunoBellardita, CarmeloMenegaz, GloriaBellido-Vallejo, José CarlosMeng, YuanBelloch-Fuster, AmparoMeo, MariannaBelmin, JoëlMestre-Francés, NadineBennett, ChristopherMetcalfe, BenjaminBenozzo, DaniloMewton, LouiseBerbrayer, DavidMeyer, Kathrin C.Bergeron, GlenMeyer, LarsBergman, LinaMeyerand, ElizabethBerkers, RuudMiddei, SilviaBerman, Brian D.Mielcarek, MichalBernstein, Hans-GertMilej, DanielBerteotti, ChiaraMileti, IlariaBerus, LucijanoMiller, Eliza CBeuriat, Pierre-AurélienMirabella, GiovanniBezzi, PaolaMishra, DushyantBiderman, MichaelMishra, Jay S.Bies, AlexanderMishra, JyotiBirkett, MelissaMitchell, Cassie S.Bisaglia, MarcoMiyazaki, TetsuroBlack, Kevin J.Mizuo, KeisukeBlack, SheilaModirrousta, MandanaBlair, ChristopherMoezzi, BaharBlair, LauraMohammadian Rad, NastaranBland, Sondra T.Molaei, SomayehBlaze, JenniferMolina-Cerrillo, JavierBlotenberg, IrisMonif, MasturaBluett, Rebecca JMontgomery, LaTriceBlum, RobertMoon, SangheeBoat, RuthMoore, Amy LawsonBogaard, AndrewMoore, EmmaBonaiuto, MarinoMoretti, RitaBosman, Conrado A.Morikawa, KazunoriBotrel, LoïcMorita-Takemura, ShokoBottini, GabriellaMorrier, Michael J.Boudewyn, Megan A.Mosienko, ValentinaBougea, AnastasiaMosley, Philip E.Bouhrara, MustaphaMou, YongchaoBoutin, JeanMueller-Oerlinghausen, BrunoBoutros, Nash N.Muhammad, YarBowler, SimonMuhsen, KhitamBowling, Heather L.Mukherjee, DibyenduBozzato, PaoloMüller, NotgerBozzi, YuriMüller, ThomasBracco, MartinaMulligan, Megan K.Brasic, JamesMur, AngelBratt, AlisonMurphy, BernadetteBraun Janzen, ThenilleMurphy, BernadetteBresin, KonradMurphy, RonanBricout, Véronique AurelieMurray, AnnaBrigham, KatharineMushtaq, FaisalBrodsky, BethMutanen, Tuomas P.Brown, Angus M.Naik, Ganesh RBrown, BelindaNaik, Ganesh R.Brown, Matt J.N.Nakayama, HaruoBrugarolas, PedroNapierala, MarekBruno, JenniferNarayana, ShaliniBryan, Robert MNarayanan, Nandakumar S.Brysbaert, MarcNaruniec, JacekBuchet, ReneNarzisi, AntonioBuján, AnaNataf, SergeBultena, SybrineNation, DanielBunford, NóraNavarro-López, Juan D.Buonaguro, Elisabetta FilomenaNelson, AimeeBurghardt, NeshaNelson, RolfBurgos-Ramos, EmmaNeuhaus, Ain A.Burke, Andrew RobertNeumann, EwaldBurkhouse, Katie L.Newman, LouiseBurugupally, Sindhu PreethamN’Gouemo, ProsperButler, Erin E.Nguyen, Khue VuButton, DuaneNiazi, Imran KhanCabib, SimonaNiazi, Imran KhanCachia, ArnaudNielsen, Boye SchnackCaffò, AlessandroNiermeyer, SusanCaillaud, MarieNieto-Diaz, ManuelCallegari, CamillaNishi, MayumiCampagna, SaraNixon, Sara JoCampolongo, PatriziaNizhnikov, Michael ECandiani, SimonaNjus, DavidCao, HengyiNoll-Hussong, MichaelCapal, Jamie K.Nordin, Andrew D.Cardoso, Fernanda CaldasNowaczewska, MagdalenaCarvelli, LuciaO’Carroll, Simon J.Castren, MaijaOh, HyukCastro, Daniel CÖhrfelt, AnnikaCauli, OmarOjetti, VeronicaCavazza, MarcOKADA, TAKESHICejudo, JavierOklu, RahmiChahrour, MariaOlivares-Olivares, Pablo JoséChakraborty, ArijitOliver, DavidChakravarthy, KrishnanOlsen, JørnChalah, Moussa AntoineOlsen, Michelle L.Chalah, Moussa AntoineOng, Jia HoongChang, Huiyi HarrietOng, Lin KooiChang, Ke-VinO’Reilly, Kally C.Charidimou, AntreasOrtner, RupertChaudhary, UjwalOsier, NicoChen, Chiung-MeiOsuagwu, BethelChen, XiPaap, Kenneth R.Cheng, JiaPack, Allan I.Cheong, Jae HoonPaetzold, Ramona L.Cheung, Po-YinPAL, AJAYChhatbar, PratikPal, Gian D.Chiappini, StefaniaPalermo, LianaChien, Jung HungPalmiero, MassimilianoChirwa, Anika S.Palotas, AndrasChoo, Pei LingPalsamy, PeriyasamyChou, Wen-HaiPankratov, YuriyChristiansen, LassePankratova, StanislavaChristie, AnitaPapousek, IlonaChroust, Alyson J.Park, EugeneChua, Koon Jiew EriPark, JaeyongChung, Moo K.Parks, ColleenChung, Tae NyoungParrella, EdoardoCinar, EyupParry-Jones, AdrianClarke, Karen-AnnParsons, TravisCocozza, SirioPastor, DiegoCoenen, MaraikePastor, JesúsCoenen, Volker A.Patel, AmarCohen, DanielPatel, HardikkumarCollier, Dami AderonkePater, LukeColombi, CostanzaPati, DipaConstable, PaulPaul, FriedemannConstantini, ShlomiPearce, Alan J.Conti, EugeniaPeckham, Alyssa M.Conversi, DavidPedapati, ErnestCooper, RobinPeles, EinatCooper-Knock, JohnathanPellicciari, Maria ConcettaCoppedè, FabioPeltola, Mikko J.Corcos, Daniel M.Penner, LouisCordero-Grande, LucilioPennington, CatherineCorrales Serrano, MarioPennington, CatherineCorrochano, SilviaPeristeri, EleniCouch, YvonnePerna, Dr. SimoneCoull, BrucePerrey, StéphaneCox, CharlesPersson, EmilCox, Christopher R.Phelix, Clyde F.Crittenden, Jill R.Piallat, BrigitteCroce, PierpaoloPiccardi, LauraCrosse, MichaelPierce, DwightCurry, TommyPignataro, GiuseppeCushing, Sharon L.Pihl-Thingvad, JesperCzosnyka, MarekPikija, SlavenD. Patterson, JohnPina, MelanieDaher, João P. L.Pini, Luigi AlbertoDahm, Stephan F.Piscopo, PaolaDaikoku, TatsuyaPitteri, MarcoDale, DeutschPlatt, Donna M.Dalmaijer, Edwin S.Pockett, SusanDaniel, Da SilvaPollak, YehudaDanisa, Olumide A.Polverino De Laureto, PatriziaDaros, AlexanderPorcaro, CamilloDas, Bhaskar CPortacolone, ElenaDasgupta, MonidipaPoston, BrachD’Atri, AuroraPower, KevinDatta, Anita N.Price, MennaDavelaar, Eddy J.Prior, SarahDavies, DavidPrough, Donald S.D’Avossa, GiovanniPushpanathan, MariaDe Bettencourt, Megan T.Pyott, Sonja J.De Bruin, AngelaQuarantelli, MarioDe La Torre, Gabriel G.Quattrocchi, Carlo CosimoDe Pascalis, VilfredoQuide, YannDe Pauw, JokeQuinlan, Leo R.De Ridder, DirkRadziminska, AgnieszkaDelgado, Montserrat G.Raffa, GiovanniDeligianni, FaniRahmathulla, GazanfarDeLuca, VincentRaikes, AdamDembek, TillRajaraman, SivaramakrishnanDening, TomRajpurohit, SurendraDe’Sperati, ClaudioRaposio, EdoardoDeutsch, DianaRaven, MelissaDewey, Rebecca SusanRedifer, JenniDey, AdwitiaReed, William R.Di Michele, LoretaReeke, George N.Diaz, AnjoliiReeves, AdamDinwiddie, Darrell L.Regenhardt, Robert WDomellöf, Magdalena ErikssonRegenold, WilliamDonofry, ShannonReitzel, JohnDöppner, Thorsten R.Rejer, IzabelaDörr, StefanRen, QingzhongDorrance, Anne M.Repetto, ClaudiaDragoi, GeorgeReschke, Cristina RDragomir, AndreiReschke, PeterDrane, PatrickReybrouck, MarkDrott, JennyReznik, Jacqueline E.Drum, ScottRhee, JoohyunDuchateau, NicolasRhodes, Justin S.Dudok, JeroenRiboldi, Giulietta M.Dugger, BrittanyRicci, LorenzoDumont, JulieRittman, TimothyDuquette, PierreRivera, VictorDyakin, Victor V.Rizk, Elias B.Dyke, KatherineRobin, JessicaDylman, AlexandraRocha Ferreira, EridanEdelsbrunner, PeterRočka, SauliusEdmonds, EmilyRodriguez, Francisca SEdwards, Adam B.Rodriguez-Contreras, AdrianEggleston, Jeffrey D.Rodríguez-Pallares, JannetteEiges, RachelRodriguez-Revenga, LaiaEl Haj, MohamadRomani, MassimoElhaik, EranRoso, Margarita CorominasElisa, KallioniemiRossi, EleonoraEllis, GeorgeRotermund, DavidFabio, Rosa A.Rothermich, KathrinFacchin, AlessioRouillard, JoséFalla, MarikaRubina, KseniyaFallacara, Dawn M.Rudroff, ThorstenFan, FanRuffino, CéliaFarioli-Vecchioli, StefanoRuge, DianeFehr, ThorstenRulseh, Aaron MichaelFeng, JingRumney, PeterFerini-Strambi, LuigiRuotolo, FrancescoFerlazzo, EdoardoRupprecht, SilkeFernandez-Vargas, JacoboRůžička, JiříFerreira, Paulo A.Rybczyńska-Tkaczyk, KamilaFillmore, PaulRyu, Ji-wonFinck, Brian N.Saba, LauraFinkel, Alan G.Sacheli, Lucia MariaFisher, CarlSacrey, Lori-Ann R.FitzGerald, JamesSadat, JasminFlak, Jonathan NSadegh-Zadeh, Seyed-AliFlanagan, ShawnSafaai, HoumanFomenko, AntonSagredo, OnintzaFoster, Jeffrey L.Sah, NirnathFrancis, HaneSaleh, SohaFranzese, AdrianaSalido, JesúsFreeman, AlanSalm, Adrienne K.Freund, NadjaSalman, MootazFriedenberg, JaySalta, EvgeniaFriedrich, ClaudiaSalvatore, JessicaFrischkorn, GidonSamadi, Sayyed AliFroriep, Ulrich P.Samadi, Sayyed AliFrykholm, PeterSammler, DanielaFuemmeler, Bernard F.Sanguinetti, JayFurmańczyk, KonradSankarasubramanian, VishwanathFysh, Matthew C.Santiago-Rodríguez, EfraínGadhoumi, KaisSarkar, ChandraniGaier, EricSarubbo, Maria FiorellaGaleazzi, Gian MariaSase, AjinkyaGaligniana, MarioSase, AjinkyaGalli, ManuelaSato, HaruhikoGambaruto, AlbertoSato, MarikoGambetti, ElisaSauer, MarkusGandola, MartinaSaul, DominikGantz, StephanieSavarese, AntoniaGao, SiyuanSchaefer, MichaelGardner, Paul D.Scheuerle, Angela E.Garibotto, ValentinaSchindler-Ivens, SheilaGarton, FleurSchmidt, EdwardGatzinsky, KlimentSchmidt, Timo TorstenGee, DylanSchmitt, AndreaGeminiani, AliceScholkmann, FelixGeorgiopoulos, CharalamposScholkmann, FelixGhag, GauravSchor, Nina F.Gilbert, FrédéricSchrier, RachelGilmore, CaseySchubert, Anna-LenaGlize, BertrandSchumann, AickoGobert, DeniseSchur, Peter H.Godler, David ESeasholtz, AudreyGoldenberg, AliceSebastianelli, LucaGómez-Anson, BeatrizSebastiano, Davide RossiGómez-Miñambres, JoaquínSeidl, AmandaGomez-Pilar, JavierSeiler, AlexanderGonçalves, Ana RibeiroSellers, KristinGonzález, Jose LuisSemendeferi, KaterinaGonzalez-Escamilla, GabrielSemple, Bridgette D.Goodie, AdamSerafini, GianlucaGorska-Ponikowska, MagdalenaSerefko, AnnaGospodarev, VadimSerniclaes, WillyGoswami, NanduServello, DomenicoGray, Joshua C.Shafai, FakhriGray, StevenShapiro, AngelaGreen, Lara ASharaev, MaksimGreengard, Emily G.Sharma, RishiGreenhill, Laurence L.Sharman, RachelGreening, StevenSheerin, Christina MGriesmaier, ElkeSheldon, RolloGrimm, Marcus OWShelley-Tremblay, JohnGriñán-Ferré, ChristianShen, GuofangGroen, Margriet ASherratt, R. SimonGrundy, LukeShi, JieGu, FengShibata, YasushiGu, YianShintel, HadasGuediche, SaraShirakawa, TetsuoGuiraudie-Capraz, GaelleShiwaku, HirokiHaarbauer-Krupa, JulietShoffstall, AndrewHabib Perez, OlindaShuffrey, Lauren C.Hackett, MarkSidiropoulos, ChristosHackney, Madeleine E.Silva, SusanaHaddow, LewisSinex, Donal G.Haining, Robert L.Singh, AmardeepHajjar, EmilySingh, ArunHalaris, AngelosSinitsyn, Dmitry O.Hall, F. ScottSipos, KatalinHalpern, AndreaSkrzypiec, GraceHamacher, DennisSłomka, ArturHamann, MartineSmith, AnnHammond, Donna L.Smith, Carolyn BeebeHampson, David R.Smith, Jared GHanas, JaySnider, SarahHand, Ivan L.Snijders, TinekeHarper, JeremySoekadar, SurjoHarrigan, Mark R.Solis-Escalante, TeodoroHashemi, AliSong, YinchenHategan, AnaSpinner, ChristophHatkevich, ClaireSquillaro, TizianaHayden, Melvin R. PeteSrinivas, UmamaheshHazrati, MehrnazSrivastava, PranayHe, YiSrivastava, SaurabhHeffner, Christopher CSt Clair-Thompson, HelenHeinbockel, ThomasStapleton-Kotloski, Jennifer R.Helminen, Terhi M.Stavrou, VasileiosHenderson, EmilySteel, KylieHenrich, KarenSteele, Vaughn R.Hentall, IanStefanis, LeonidasHernan, AmandaŠtefulj, JasminkaHerrera, FedericoStegemoller, ElizabethHervás-Marin, DavidStenz, LudwigHeuer, AnnaStocker, RetoHeyn, PatriciaStraburzynski, MarcinHidalgo, JuanStraßer, TorstenHigashida, HaruhiroStrutton, Paul H.Higuchi, YoshinoriSubramaniyan, ManivannanHilbert, SvenSujobert, PierreHilhorst, MarcSullivan, Kelly M.Hills, Peter J.Summers, RebekahHilton, ClaudiaSun, NaweiHiraoka, KoichiSun, WoongHoda, MD NasrulSupek, SelmaHolleran, Katherine MSurguchov, AndreiHordacre, BrentonSurguchov, AndreiHori, HikaruSuyama, KeitaroHorn, SebastianSyaekhoni, M. AlexHosford, Patrick SSzabo-Reed, Amanda N.Hoshi, AkihikoSzatmari, ErzsebetHowell, BryanSzczepek, Agnieszka J.Huang, Helen J.Szigeti, KingaHulleman, JohanSzumlinski, KarenHunter, Carla Desi-AnnTabar, Yousef RezaeiHutsler, JeffreyTabolacci, ElisabettaHüttemann, MaikTagu, JérômeHyde, ChristianTajadura-Jimenez, AnaHyland Bruno, JuliaTajik-Parvinchi, DianaIannilli, EmiliaTakahashi, TetsuyaIchimura, ShinyaTakano, KatsuraIga, JunichiTakuma, KazuhiroIgloi, KingaTames, JoseIgnacio Arribas, JuanTan, LeeIguchi, TaisenTanaka, NaoroIhle, AndreasTang, ShiyuImamura, FumiakiTarantini, StefanoImani, MahdiTassé, MarcIndahlastari, AprindaTatsumi, KoukoIneichen, ChristianTaylor, GemmaInta, DragosTeh, RuthInvitto, SaraTeixeira, CésarIon, IoanaTen Brink, Antonia F.Ipser, AlbertaThayyil, SudhinIsraeli-Korn, SimonThomas Tobin, CourtneyJ, Gil-MohapelThomas, KelseyJacova, ClaudiaThompson, BarbaraJain, KartikToepel, UlrikeJain, SameerTomporowski, PhillipJaiswal, Ashvin R.Tonetti, LorenzoJakovcevski, MiraTönges, LarsJangampalli Adi, PradeepkiranToraldo, AlessioJanigro, DamirTorregrossam, MaryJanjua, M. BurhanTorres Filho, Ivo P.Jaquess, KyleTorres, NapoleonJaschke, Artur CTourjman, Smadar ValérieJendelova, PavlaTrenado, CarlosJentschke, SebastianTrewartha, KevinJeong, Keun-YeongTripi, GabrieleJerin, AlešTrue, LarissaJerzemowska, GrażynaTrujillo, Logan T.Jesulola, EmmanuelTsapanou, AngelikiJiang, ChengTsukamoto, TetsuoJiang, JianxiongTsytsarev, VassiliyJiménez-Bonilla, Julio FranciscoTurner, Arlener D.Joffe, Max ETurpin, NicolasJóhannesson, Ómar I.Uchino, HarutoJohnson, JeffUddin, ZakirJohnson, SheriUhl, EberhardJones, LesleyUnterrainer, JosefJu, GaoUsdin, KarenJurjans, KristapsVaibhav, KumarJurkowlaniec, EdytaVan De Ven, Marco Jusas, VaciusVan Eck, RichardJutzeler, Catherine J.Van Roon, Willeke M.C.Kajiwara, YusukeVan ’t Wout, MaschaKalkman, Hans OVan Veen, Teelkien RKalmar, JayneVan Vugt, MariekeKane, CynthiaVance, David E.Kang, SinyoungVellante, FedericaKanne, Stephen M.Vergo, Maxwell TKarim, AhmedVerschuuren, JanKarim, NasiaraVersnel, HuibKarkhanis, AnushreeVitek, MichaelKarlsson, PetraVoderholzer, UlrichKarmakar, Chandan KumarVoellger, BenjaminKastrup, BernardoVoltas, NuriaKataki, MariaVulchanova, Mila DimitrovaKatrinli, SeymaWaheed, WaqarKatsila, TheodoraWais, Peter E.Kayed, RakezWalia, Seema G.Kelemen, William L.Wallace, Douglas M.Kelly, DavidWang, XinkunKemp, KevinWang, YinaKennedy, DavidWard, JamieKernick, DavidWarmington, MeeshaKerzel, DirkWarren, ChrisKhan, NaimulWarren, Steven F.Khazipov, RoustemWaser, MarkusKhokhar, BilalWashizawa, YoshikazuKillen, MelanieWatanabe, EiichiKilliany, Ronald J.Watkins, Laura E.Kim, Hyung-JunWessely, SimonKim, Yeo HyungWestmark, CaraKim-Mitsuyama, ShokeiWężyk, MichalinaKindermann, HaraldWhitaker-Azmitia, Patricia M.Kintzelé, LaurentWhittaker, Joseph R.Kious, BrentWiese, RichardKireev, MaximWilf, MeytalKnight, HelenWilker, SarahKnowlton, Barbara J.Williams, KimberlyKoch, KathrinWilloughby, LouisaKodali, MaheedharWimmer, MathieuKoelsch, StefanWinner, BeateKoike, TakahikoWirick, SueKoike, YasuharuWlaź, PiotrKoleňák, JiříWolstenholme, JenniferKornhuber, Malte.Wolter, TilmanKORTAGERE, SANDHYAWu, Chun-HuKotchoubey, BorisWu, Monica S.Kothgassner, Oswald DavidXi, ZhengxiongKoyama, RyutaXie, RuiliKraus, ChristophXie, ShaneKretzschmar, AndréXiong, LanKrueger, JoachimXu, WilliamKrysko, KristenXu, XinKrzystanek, MarekYamamotova, AnnaKrzyżewski, Roger MYang, JialeKukull, Walter A.Yang, XueyingKumar, AmitYano, HirohitoKumar, GauravYates, Nathanael J.Kwakowsky, AndreaYe, XinLa Foresta, FabioYegla, Brittney A.Laezza, FernandaYen, Cheng-FangLagarde, EmmanuelYielder, Paul C.Lago, Tiffany R.Yin, JunLaguna, AriadnaYong, Kar WeyLahmiri, SalimYoun, Young ChulLahmiri, SalimYoung, Katherine S.Lai, YanhaoYoung, KymberlyLangeard, AntoineYoussof Zadeh, VahabLanza, GiuseppeYunusa, IsmaeelLardone, AnnaZähle, TinoLaurin, JérômeZaitsev, AlekseyLawrence, KatherineZani, AlbertoLe Merrer, JulieZapico, Sara C.Leasure, J. LeighZeid, OmarLebon, FlorentZetterberg, HenrikLeder, JohannesZhang, JixiangLee, Darrin JasonZhang, WeiweiLee, HyekyoungZhang, YangLee, Jin-KooZhang, YangLee, Meng-JenZhang, YuLee, Wang-TsoZhao, BoxuanLees, JustinZhou, MinLeone, MassimoZille, MariettaLewis, GregoryZimmer-Bensch, GeraldineLi, HuiquanZiolkowski, WieslawLi, ShuaiZito, Giuseppe AngeloLi, YunghuiZivony, AlonLiao, YichuZomorrodi, RezaLiew, ZeyanZuber, SaschaLin, Pei-FengZurrón, MontserratLin, WeihongZvyagintsev, Mikhail

